# Luteolin and curcumin in rodent models of cognitive disorders: a dose‐response perspective

**DOI:** 10.1002/alz.092888

**Published:** 2025-01-09

**Authors:** Giulia A Führ, Aline R. Zimmer

**Affiliations:** ^1^ Federal University of Rio Grande do Sul, Porto Alegre, RS Brazil; ^2^ Federal University of Rio Grande do Sul, Porto Alegre, Rio Grande do Sul Brazil

## Abstract

**Background:**

Alzheimer’s disease (AD) is a progressive neurodegenerative disease and the most common form of dementia. Although AD is characterized by the accumulation of amyloid beta (Aβ) plaques and neurofibrillary tangles (NFTs), it’s estimated that nearly half of AD cases might be attributed to modifiable risk factors and lifestyle‐based interventions may offer promising preventative strategies to delay disease onset and progression. Polyphenolic derivatives easily found in foods like luteolin and curcumin have shown beneficial effects to counteract cognitive decline.

**Method:**

A systematic review was conducted to determine the oral therapeutic doses of curcumin and luteolin used in experimental models of cognitive disorders in rodents, presenting beneficial effects. Public databases, including PubMed, Embase, Web of Science and Scopus were searched. Original research and review papers published between 2009 and 2023 using the following keywords: “luteolin,” “curcumin,” “oral administration,” “cognitive disorder,” “mice,” and “rats” were included in the search.

**Result:**

Eleven articles for luteolin and twenty‐seven for curcumin fit the inclusion criteria. For luteolin, the oral doses range from 1.25 mg/kg to 200 mg/kg, with treatment length from 7 days to 5 months. For curcumin, oral doses range from 0.4 mg/kg to 400 mg/kg, with treatment length from 2 days to 6 months. The studies demonstrated that luteolin reduces the deposition of Aβ, oxidative stress, neuroinflammation, neuronal damage and neuronal insulin resistance, improving learning and memory and rescuing cognitive impairment. Curcumin reduces oxidative stress and Aβ production and promotes hippocampal neurogenesis, improving spatial learning, memory and cognitive function. None of the studies evaluated reported toxic effects at the tested doses. The use of both compounds in combination was not found in the literature. The most promising doses for improving cognitive function in experimental models ranging 10 – 20 mg/kg for luteolin and 25 – 50 mg/kg for curcumin.

**Conclusion:**

Luteolin and curcumin exhibit multi‐target pharmacological properties, suggesting the potential to prevent cognitive disorders, including AD. The neuroprotective effect of these agents in combination and whether this would lead to a dose reduction and an improvement in effect is still a subject to be explored.

